# Malignant mixed Mullerian tumors of the uterus: histopathological evaluation of cell cycle and apoptotic regulatory proteins

**DOI:** 10.1186/1477-7819-8-60

**Published:** 2010-07-19

**Authors:** Rani Kanthan, Jenna-Lynn B Senger, Dana Diudea

**Affiliations:** 1 Department of Pathology and Laboratory Medicine, University of Saskatchewan, Saskatoon, SK, Canada

## Abstract

**Aim:**

The aim of our study was to evaluate survival outcomes in malignant mixed Mullerian tumors (MMMT) of the uterus with respect to the role of cell cycle and apoptotic regulatory proteins in the carcinomatous and sarcomatous components.

**Methods:**

23 cases of uterine MMMT identified from the Saskatchewan Cancer Agency (1970-1999) were evaluated. Immunohistochemical expression of Bad, Mcl-1, bcl-x, bak, mdm2, bax, p16, p21, p53, p27, EMA, Bcl-2, Ki67 and PCNA was correlated with clinico-pathological data including survival outcomes.

**Results:**

Histopathological examination confirmed malignant epithelial component with homologous (12 cases) and heterologous (11 cases) sarcomatous elements. P53 was strongly expressed (70-95%) in 15 cases and negative in 5 cases. The average survival in the p53+ve cases was 3.56 years as opposed to 8.94 years in p53-ve cases. Overexpression of p16 and Mcl-1 were observed in patients with longer survival outcomes (> 2 years). P16 and p21 were overexpressed in the carcinomatous and sarcomatous elements respectively. Cyclin-D1 was focally expressed only in the carcinomatous elements.

**Conclusions:**

Our study supports that a) cell cycle and apoptotic regulatory protein dysregulation is an important pathway for tumorigenesis and b) p53 is an important immunoprognostic marker in MMMT of the uterus.

## Background

Malignant mixed Mullerian tumors (MMMT) of the uterus are rare, high-grade neoplasms comprising only 1-2% of uterine cancers [1] and 3-5% of all uterine malignancies [2]. They are the most common variety of mixed epithelial and non-epithelial endometrial tumors, with a clinically aggressive course [3,4]. Stage of the disease and the depth of myometrial invasion are recognized as important prognostic factors [5-7]. Two-year survival rates have been reported at 53% in stage I (confined to uterine corpus) and 8.5% in stages II (cervical metastases) and III (pelvic metastases), with none reported in Stage IV [8]. Common in the uterus, this tumor may arise in the ovaries, fallopian tubes and vagina [5,9]. Histologically, MMMT is a biphasic tumor composed of both epithelial (carcinoma) elements and mesenchymal (sarcoma) elements; though, which component is responsible for the tumor's aggressive biological behavior remains undetermined [2,10-15].

Three theories proposed to ascertain this tumor's histiogenesis include that MMMTs may be 1) collision tumors, 2) combination tumors, or 3) composition tumors. Immunophenotypical and ultrastructural studies that favor the third theory explain MMMTs as being monoclonal in origin, with diverse carcinomatous and sarcomatous elements that can be homologous (histologically native, worse prognosis) or heterologous (foreign, better prognosis) to the organ [13,15-18]. MMMTs occur in postmenopausal women and usually present in an advanced stage with abdominal pain, distension, and atypical spotting/bleeding [18-21]. While it is presumed that MMMTs arise from pre-existing carcinomas, little is known about the etiopathogenesis of MMMTs. Exposure to radiation, excessive estrogen exposure, obesity, and nulliparity [22,23] are believed to be associated with MMMT development.

It is usually understood that carcinogenesis is a multistep process that involves defects of the genetic pathways including cell proliferation, cell adhesion, cell death and apoptosis [2]. Cell survival and apoptotic regulatory proteins such as the Bcl-2 family of genes, PCNA, p16, p21, p27, and cyclin D1 are of vital importance to malignant neoplasms in prolonging cell survival. Despite the understanding that cell cycle regulatory protein dysregulation may be involved in numerous malignant tumors [2], there is limited data that explores the role of these oncoproteins with survival data in MMMTs. The aim of this study is to evaluate the role of cell cycle and apoptotic regulatory proteins in the carcinomatous and sarcomatous components of uterine MMMT in relation to clinico-pathological data including survival outcomes.

## Materials and methods

**Figure 1:**
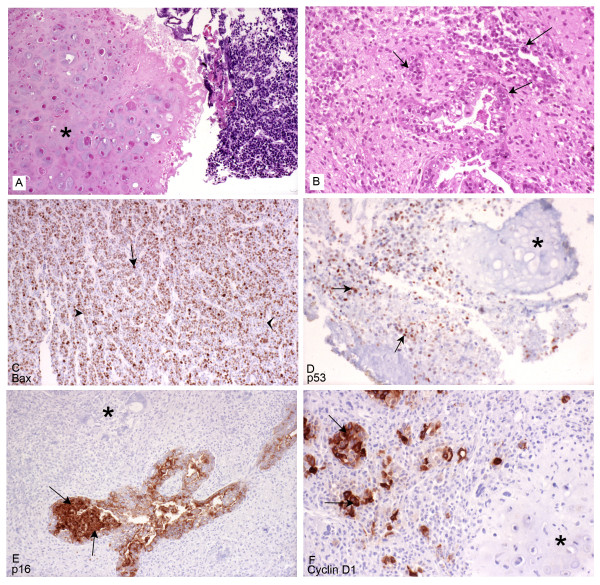
**Histopathological Evaluation with Immunohistochemical Staining**. **A: **Hematoxylin-eosin stain (original magnification ×250). The star (*) indicates the malignant heterologous component of uterine MMMT. **B: **Hematoxylin-eosin stain (original magnification ×250). The arrows indicate the malignant epithelial component of uterine MMMT. **C: **Staining with Bax antibody (original magnification ×250). The expression of Bax antibody is diffuse with the thin arrowhead indicating weak staining, the thick arrowhead indicating medium staining, and the tailed arrow indicating strong staining. **D: **Staining with p53 antibody(original magnification ×250). The star (*) indicates the negatively stained heterologous sarcomatous element, and the arrow indicates positive staining in the epithelial component. **E: **Staining with p16 antibody (original magnification ×250). The star (*) indicates the negatively stained region, and the arrow indicates the strong positive staining in the malignant epithelial glands. **F: **Staining with Cyclin D1 antibody (original magnification ×250). The star (*) indicates the negatively stained heterologous sarcomatous element, while the arrow indicates a focus positive staining in the adjacent epithelial component.

Twenty-three cases of uterine MMMT were identified from the records of the Saskatchewan Cancer Agency (1970-99). The original slides and paraffin blocks were retrieved and reviewed to confirm the diagnosis as seen in Figures 1A and 1B. A representative block was chosen for detailed histological and immunohistochemical study with the antibodies as listed in Table 1. EMA, Bcl-2, Ki67, PCNA, Bad, Mcl-1; bcl-x, bak, mdm2, bax, p16, p21, p27, p53 and Cyclin D1 expression were evaluated by the standard avidin-biotin complex method with positive and negative controls as per standard laboratory protocol. Immunostaining results were scored on a semi-quantitative scale including staining intensity and percentage of positive cells. The extent of immunostaining was divided into four categories according to the percentage of immunostained neoplastic cells: < 25% (1+), 25-50% (2+), 50-75% (3+), and > 75% (4+). In addition, the qualitative intensity of immunostaining of the tumor cells was quantitatively scored into three categories: weak (1+), moderate (2+), and strong (3+) as seen in Figure 1C. The intensity of the endothelial cell staining served as an internal control. A combined immunoreactivity score was calculated by multiplying the score for the percentage x the score of intensity, resulting in a combined score that ranged from 0 to 12. Scores 4 and above were considered positive for the purposes of this study.

**Table 1:** Clone, dilution, and source of antibodies used for the immunohistochemical analysis in this study

ANTIBODY	CLONE	DILUTION	SOURCE
EMA	Clone E29	1/800	Dako
Ki-67	Clone MIB-1	1/50	Immunotech
PCNA	Clone PC-10	N/A	Ventana
Bcl-2	Clone 124	1/20	Dako
P53	Clone DO-7	1/50	Dako
Bad	48	1/20	BD (BioSciences)
Mcl-1	38G3	1/500	Novacastra
Bcl-x	NC1	1/20	Novacastra
Mdm2	IB10	1/40	Novacastra
Bak	polyclonal	1/20	Dako
Cyclin D1	polyclonal	1/50	Dako
Bax	polyclonal	1/50	Dako
Ki67	Clone MIB-1	1/50	Immunotech
P53	Clone DO-7	1/50	Dako
P16	F-12	1/100	Santa-Cruz
P21	EA10	1/10	Oncogene
P27	SXS3G8	1/20	Dako
Cyclin D1	P2D11F11	Predilute	Ventana

Clinical data such as disease free survival, overall survival, family history of cancer, past medical history, and treatment protocols were obtained by detailed analysis of their case records. Statistical analysis was preformed with Kruskal-Wallis, Fischer's Exact Test, and a Mann-Whitney post hoc test for independent data. A *p *value of ≤ 0.05 was regarded as statistically significant.

This study was conducted with ethics approval from the University of Saskatchewan Biomedical Research Ethics Review Committee.

## Results

### Demographics and Clinical Measures

**Figure 2:**
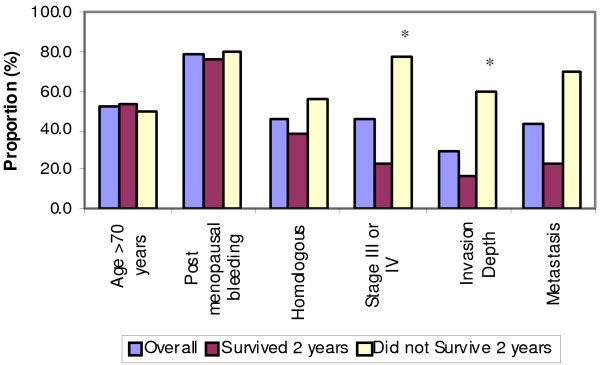
**Demographic and Clinical Data in Relation to 2 Year Survival Data**. X-axis displays: age, postmenopausal bleeding, homologous elements, stage III/IV, depth of invasion, and metastasis. Y-axis displays: survival outcome data-including overall survival (purple bars), two-year survival (maroon bars), and less than two-year survival (yellow bars). * p < 0.05 based on Fisher's Exact Test.

Table 2 and Figure 2 list the various demographic and clinico-pathological data of 23 patients with uterine MMMT. The majority of patients (39.1%) were 71-80 years old, followed by 61-70 years (26.1%). 18 patients (78.3%) presented with postmenopausal bleeding. Histologically, 11 patients (47.8%) had homologous elements, while 10 (43.5%) had heterologous elements. 10 patients (43.5%) were Stage I, two (8.7%) Stage II, three (13.0%) Stage III and seven (30.4%) Stage IV. Myometrial depth of invasion was superficial in 43.5% of patients, and deep in 30.4%. Metastases were present in 43.5% of patients at presentation in the liver, ovaries, fallopian tube, abdomen, peritoneum, ommentum, bladder, and iliac lymph nodes. Five cases (21.7%) exhibited pelvic metastasis. Lung and cervical metastasis were present in 2 patients (8.7%). Management protocols included surgery (20 patients, 87.0%), chemotherapy, (2 patients, 8.7%) and radiation therapy (14 patients, 60.9%).

**Table 2:** Patient demographics with clinico-pathological data

Category	Number of cases	Percentage (%)
**Age group**		
50-60 years	5	21.7
61-70 years	6	26.1
71-80 years	9	39.1
81-90 years	3	13.0
**Postmenopausal bleeding**		
Yes	18	78.3
No	5	21.7
**Histological type**		
Homologous	11	47.8
Heterologous	10	43.5
**Stage**		
I	10	43.5
II	2	8.7
III	3	13.0
IV	7	30.4
**Depth of invasion**		
Superficial	10	43.5
Deep	7	30.4
**Metastasis**		
Any	18	78.3
Lung	2	8.7
Pelvic	5	21.7
Cervical	2	8.7
Other	9	39.1
**Treatment**		
Surgery	20	87.0
Chemotherapy	2	8.7
Radiation	14	60.9
Other	8	34.8

The number of cases and their percentage are listed with respect to age group, postmenopausal bleeding, histological type, stage, depth of invasion, metastases, and treatment modalities.

### Relationship with Survival Time

One of the main goals of this study was to determine the prognostic value of the demographic and clinico-pathological data to the immunohistochemical expression of the biological markers studied. The stage of the disease (Table 2 and Figure 2) indicates the proportion of subjects surviving 2-years. Of the 23 cases, 3 (13.0%) were Stage III, and 7 (30.4%) Stage IV. 77.8% of patients in Stage III/IV survived less than two years while 23.1% had longer survival outcomes. This inference of survival data may not be truly reflective as treatment protocols were not standardized across all cases studied.

**Figure 3:**
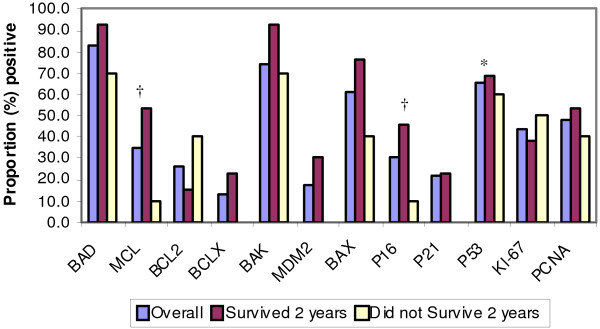
**Cell Cycle and Apoptotic Regulatory Proteins in Relation to 2 Year Survival Data**. Statistical significance: *p < 0.05, †p < 0.10.

Cell cycle and apoptotic regulatory proteins were analyzed for statistical significance as possible prognostic indicators. Two cell cycle proteins, Mcl-1 and p16, were found to be statistically significant. Of the 23 patients, 8 (34.8%) were positive for Mcl-1. As seen in Figure 3, survivors of 2 years, 53.8% had a positive Mcl-1 expression, while only 10.0% of those cases that did not survive showed positive Mcl-1 expression. Similar results were obtained for p16. Seven cases (30.4%) were positive for this cell cycle protein. Of the patients who survived two years, 46.2% exhibited positive p16 expression, while only 10.0% of those who did not survive 2 years did. P53 was strongly expressed (70-95%) in 15 cases and negative in 5 cases. The average survival in the P53+ve cases was 3.56 years as opposed to 8.94 years in P53-ve cases.

### Protein Markers

The histological samples from these 23 cases were analyzed for the presence of various biological markers, including both cell cycle and apoptotic regulatory proteins. The cell cycle proteins studied include p16, p21, p27, p53, Cyclin D1 and Ki67. As seen in Table 3, p16 was positively expressed in 7 cases (30.4%), p53 in 15 cases (65.2%), and Ki67 in 10 patients (43.5%). There was no overexpression of p27. Cyclin D1, though predominantly negative, was expressed focally in the carcinomatous elements.

**Table 3:** Immunohistochemical expression of cell cycle and apoptotic regulatory protein antibodies

Antibody Used	Number of Cases	% Positive
BAD	19	82.6
Mcl-1	8	34.8
Bcl-2	6	26.1
Bcl-x	3	13.0
Bak	17	73.9
Mdm-2	4	17.4
Bax	14	60.9
p16	7	30.4
p21	5	21.7
p27	0	0
p53	15	65.2
Cyc-D1	0	0
Ki-67	10	43.5
PCNA	11	47.8

The number of cases and their percentage of expression are listed with respect to the oncoproteins used in this study: Bad, Mcl-1, Bcl-2, Bcl-x, Bak, Mdm-2, Bax, p16, p21, p27, p53, Cyc-D1, Ki-67, PCNA.

The apoptotic regulatory proteins, which were analyzed, include the following: Bad, Bak, Mcl-1, Bcl-2, Bcl-x, Mdm-2, and Bax. Bad was overexpressed in 82.6% of cases (19 patients) and Bak was positive in 73.9% (17 patients). Positive Bax expression was seen in 60.9% (14 patients). The remaining proteins had less than 50% positive expression (Figure 3).

## Discussion

Uterine MMMT are malignant neoplasms that contain atypical malignant endometrial glands admixed with heterologous or homologous sarcomatous elements [2,10-14] with the dominant element often being the epithelial component yet distinct from endometrial carcinoma[13]. Occurring predominantly in postmenopausal women [24,25], the prognosis of MMMT is generally worse than endometrial carcinoma. These are rare tumors with an annual incidence of 2/100 000 women, comprising 2-5% of all gynecologic tumors [26,27]. Five-year survival rates are reported between 18-39%. Many cases (70%) present with advanced disease (Stage III/IV), contributing to poor survival rates [21]. This tumor spreads locally within the pelvic cavity and distally to the regional lymph nodes, lungs and liver. DiSaia *et al. *[28] reported a 2 year survival rate of 53% in patients with tumors confined to the uterine corpus (Stage I), which dropped to 8.5% if the disease had extended into the cervix, vagina or parametrium (Stages II/IIII). Less than two year survival was seen in Stage IV disease [4], similar to other studies, with 5-year disease-free survival rates being: Stage 1 56%, Stage II 31%, Stage III 13%, Stage IV 0% [29]. Our study revealed similar trends. Three cases diagnosed as Stage III did not survive beyond two years. 44.4% of seven cases diagnosed as Stage IV did not survive beyond two years. However, three stage IV patients had longer survival outcomes in contrast to published literature [5]. This finding may be related to small sample size.

The stage of the disease and the depth of myometrial invasion were statistically significant prognostic factors in our study, similar to reports by other authors [5,7]. Other demographic and clinico-pathological data including age, postmenopausal bleeding, histological type, metastasis, and treatment modalities were not found to be statistically significant in our study.

Uterine MMMT metastasizes similar to endometrial carcinoma of the uterus, with recurrence occurring commonly in the upper abdomen with occasional distant spread [28]. In our study, 43.5% developed some form of metastasis. Metastases occurred in 70.0% of subjects who did not survive 2 years while longer survival time was associated with lowered metastases (23.1%). This is statistically significant, and indicates that the presence of metastasis at presentation is a strong prognostic indicator for overall survival outcomes. The exact nature of whether the carcinomatous or the sarcomatous element is the more aggressive component and therefore has greater propensity for metastases remains an unresolved and controversial issue [13].

Uterine MMMTs consist of carcinomatous (CA) and sarcomatous components (SA). Histopathological evaluation of which component is responsible for biological tumor aggressiveness has not been greatly explored. Yoshida et al [8] reported a higher microvessel density in the carcinomatous region and a higher apoptotic index in the sarcomatous areas, from which they concluded that the carcinomatous components likely played an important role in the aggressive biological behavior of MMMT [22]. This biological behavior is similar to endometrial carcinoma with which they share common etiopathological factors.

Cell proliferation, including initiation, promotion and progression (invasion and metastasis), plays a central role in the multistep process of carcinogenesis. Replication of damaged DNA is necessary to fix base substitutions, frame shift mutations, allelic deletion and induction of chromosomal translocations. Cancer cells commonly demonstrate errors in these pathways during cell cycle proliferation. Proliferative markers such as Ki67 provide an index of cells in the Go/G1 pool of cycling cells [30]. Higher fractions of these cells represent an increased number of cells subjected to genetic instability. In our study Ki67 was overexpressed (50-80% positive cells) in 10 cases with no statistical difference between the carcinomatous and the sarcomatous areas. This lack of difference in antigen expression between the epithelial and the sarcomatous areas is consistent with other studies [2]; thus supporting that the histogenesis of this tumor is probably from a single pluripotential malignant clone with divergent histologic differentiation [2].

Mutations in the *p53 *gene (tumor suppresser and gatekeeper) remain one of the commonest genetic lesions found in human cancers. This occurs in both the carcinomatous and sarcomatous elements of uterine MMMT [31-35]. Such mutations result in abnormal protein expression, with increased intracellular accumulation due to an increased half life which is easily detected by immunohistochemical methods [36-38]. In our series, overexpression of p53 (70-95%) was negative in 8 cases and positive in 15 cases, predominantly in the sarcomatous regions as seen in Figure 1D. The average survival time in p53 positive cases was 3.56 years as opposed to 8.94 years in the negatively stained cases. The cohort of the positive cases was also predominantly older (71-90 years). Though p53 positive expression cases in our study were not statistically significant in regard to survival beyond two years, it was interesting to note that p53 negative cases were associated with an improved clinical outcome. Overexpression of p53 has been linked to decreased survival in several other malignancies. These include human soft tissue sarcomas [39] and some cases of breast, lung and colorectal carcinomas [40]. Such trends clearly suggest that p53 may play a key role in the multistep evolution of disease progression in MMMT [41,42]; however, it is postulated like in pulmonary carcinosarcoma to be a late event in the disease progression with resultant better survival outcomes in those cases that have not yet acquired the defect [43]. In this context, further studies of p53 mutation analysis by PCR-SSCP with sequencing will help confirm these observed trends.

Central to the cell cycle regulatory protein machinery is a family of serine-threonine kinases, the cyclin dependent kinases (CDKs). These kinases are activated by cyclins D and E and inactivated by CDK inhibitors (CDKIs) including: p27, p16, and p21[44].

*p16 *specifically inhibits the cyclin D1-CDK4/6 complex and, along with the main substrate, forms the retinoblastoma gene product (pRb), which is the most important regulatory pathway involved in the G1/S transition [45,46]. Frequent expression of p16 in primary tumors suggests that the p16 protein is involved in the development of these lesions [2]. Uncontrolled tumor cell proliferation is frequent in tumor cells with the progression of a normal cell to a transformed tumor cell involving many genetic events that include the checkpoints of the cell cycle machinery [44,47]. Overexpression of p16 is believed to be the result of mutated p16 gene product and/or an accumulation due to decreased turnover of the protein [2]. Overexpression of p16 in the carcinomatous regions of MMMT (Figure 1E) with inverse expression of p21 in these regions denotes an upregulation of p16. The latter is also supported by a failure to express cyclin D1 in the majority of the tumor cells with focal expression only in the carcinomatous elements (Figure 1F). This supports the theory of a damaged regulatory pathway wherein p16 predominantly inhibits cyclin-D1 associated kinase activities [48]. P16 could also mediate contact inhibition of growth and thus may be responsible for the invasive powers of the neoplasm. It is interesting to note that many of the initial metastases in MMMT consist entirely of carcinomatous elements, thus supporting the theory that the carcinomatous component is perhaps responsible for the initial biological aggressiveness of the tumor. This change over time is also reported in the literature as loss of p16 in some cases of MMMTs when they recurred [2]. In our study cases 46.2% of subjects who survived 2 years had positive p16 overexpression in contrast to a lower expression in 10% of cases with less than two year survival.

Cell death plays an important role in normal tissue homeostasis wherein the finite balance between new cell productions caused by cell division is offset by cell loss in tissues capable of cell renewal. Cells that succumb to this mechanism of cell death undergo characteristic morphological and biochemical changes that are termed apoptosis. Apoptosis is one aspect of mammalian cell behavior, which is of central importance in growth and development and plays a key role in tumor-oncogenesis. The three key features of apoptosis and cell survival are related to inciting the signal transduction pathways of the bcl-2 family of genes and the ICE family of proteases. These components interact with other cell cycle related genes such as p53. The central role of the Bcl-2 family of genes in the regulation of apoptosis has been convincingly demonstrated [49-56]. The interaction of Bcl-2 family of proteins is viewed in terms of two mechanisms: a) at least two rheostats - the Bcl-2/Bax ratio and the Bcl-x_L_/Bcl-x_s _ratio and b) a quaternary complex involving anti-apoptotic protein, pro-apoptotic protein, caspase and Apf-1 equivalent protein. The susceptibility to apoptosis is likely to be determined by the ratio of the positive regulators (Bak, Bax, Bcl-x_s_) to negative regulators (Bcl-2, Bcl-x_L_, Mcl-1 and A1) [57]. The role and contribution of each of these factors is likely to be specific for different cells and tissues. The function of Bcl-2 protein is dependent on post-translational modification, specifically phosphorylation of serine/threonine residues.

Therefore, mere overexpression of the protein does not provide complete information. Also, the finding that Bcl-2 is not expressed in a variety of tumors indicates that other apoptosis-modulating factors, especially Bcl-x_L_/Bcl-x_s_, may play a role [58-63]. In view of the dimeric interactions of Bcl-2 family proteins and interaction with other apoptosis regulators, assessment of one protein alone is unlikely to provide an understanding of apoptosis regulation. Deregulation of the biochemical pathways that control physiological cell death can contribute to neoplastic cell expansion by preventing or delaying normal cell death. One of the critical regulators of apoptosis is the protein encoded by the Bcl-2 gene [64,65]. Although the exact biochemical mechanism of Bcl-2 remains enigmatic, the Bcl-2 protein appears to control a distal step in the final common pathway for apoptotic cell death. Recently, a family of genes have been identified whose encoded proteins share amino acid sequence homology with Bcl-2. Some of these genes function as blockers of cell death while others promote apoptosis [56,57,66]. Among this multigene family, the protein encoded by the Bax gene has emerged as a central regulator [65,67,68]. The Bax protein is a promoter of cell death, while others such as Bcl-x and Mcl-1 function as suppressors of cell death. Further, it has been proposed that the relative sensitivity of cells to apoptotic stimuli is governed by the ratio of Bax: Bcl-2 and other antiapoptotic Bcl-2 family proteins [58]. Gene transfer experiments indicate that Bax is a regulator, not an effector of the programmed cell death pathway. As a result, it should be possible to induce apoptosis even in the absence of Bax provided that the apoptosis stimulus is of sufficient strength. Since Bcl-2 can abrogate apoptosis promoted by Bax, it is possible that it is Bax that mainly regulates the threshold of loss of apoptosis. P53 is known to regulate Bax expression, with inactivation of p53 leading to reduced Bax protein levels [69,70]. Bax mutations and resistance to apoptosis have been described in stomach, pancreas, endometrium, hemopoietic malignancies, and a subset of colon and lung cancers [71,72] indicating that inactivating Bax mutations may play an important role in tumor progression in these cancers.

In our study, all cases demonstrated diffuse expression of Bax, Bad and Bak proteins in contrast to weak or negative expression of Mcl-1, MDM2 and Bcl-x. This supports the existence of apoptosis protein dysregulation in these lesions. The exact biochemical mechanism of such dysregulatory pathways remains unclear. Expression of Mcl-1 was not statistically significant in regard to the two year survival data; though, Mcl-1 was expressed in a higher proportion of cases that survived 2 years. This finding needs further investigation in larger samples.

Currently, there is no consensus treatment guidelines related to improved survival outcomes. The rarity of this tumor limits the potential for large clinical trials [73]. Nevertheless, the persistent high mortality rate and high recurrence rate [73] with no significant improvement in patient survival during the past 40 years [24] demands the attention and time of researchers in a fight to improve treatment modalities and widen the understanding of uterine MMMT.

## Conclusions

Our study supports that cell cycle and apoptotic regulatory protein dysregulation is an important pathway for tumorigenesis. Apoptotic protein dysregulation may result in epigenetic silencing of cell cycle pathways resulting in disarrayed/differential growth patterns. Future genetic analysis of Bad/bax/bak pathway may provide further insight in elucidating this mechanism.

In uterine MMMT p53+ve tumors occur in older women with a short mean survival while p53-ve tumors occur in younger women with longer survival. Such trends suggest that p53 may be an important immunoprognostic marker in this neoplasm that warrants further exploration.

Both p16 and Mcl-1 expression were associated with longer survival (>2 years). Further research regarding these cell cycle regulatory proteins will shed light into their possibility as future predictive/prognostic markers.

## Competing interests

The authors declare that they have no competing interests.

## Authors' contributions

RK is the corresponding, and first author of this manuscript. JLBS contributed to the acquisition, analysis, and interpretation of data. DD provided overall expertise. All authors have read and approved the final manuscript.
